# Murine typhus is a common cause of acute febrile illness in Bandung, Indonesia

**DOI:** 10.1371/journal.pone.0283135

**Published:** 2023-07-07

**Authors:** Silvita Fitri Riswari, Susantina Prodjosoewojo, Siti Rasnawati Mony, Imam Megantara, Shelly Iskandar, Wulan Mayasari, Henhen Heryaman, Quirijn de Mast, Andre van der Ven, Herman Kosasih, Bachti Alisjahbana

**Affiliations:** 1 Department of Biomedical Sciences, Faculty of Medicine, Universitas Padjadjaran, Bandung, Indonesia; 2 Research Center for Care and Control of Infectious Diseases (RC3ID), Universitas Padjadjaran, Bandung, Indonesia; 3 Department of Internal Medicine and the Radboud Center for Infectious Diseases, Radboud University Medical Center, Nijmegen, The Netherlands; 4 Department of Internal Medicine, Hasan Sadikin General Hospital, Bandung, Indonesia; 5 Department of Psychiatry, Hasan Sadikin General Hospital, Bandung, Indonesia; 6 Indonesia Research Partnership on Infectious Diseases (INA-RESPOND), Jakarta, Indonesia; Universitas Syiah Kuala, INDONESIA

## Abstract

Murine typhus (MT), an infection caused by the gram-negative bacteria *Rickettsia typhi* (*R*. *typhi*), is a significant cause of acute febrile illness (AFI) in Southeast Asia but is rarely reported in Indonesia. The current study aimed to describe the clinical characteristics of MT cases in Bandung, West Java. Non-confirmed AFI cases (n = 176) from a prospective cohort study of whom paired serum samples (acute (T1), midterm (T2), or convalescent (T3)) were available were screened using MT serology. IgG against *R*. *typhi* was detected in the T2 or T3 samples using an in-house ELISA. Positive IgG samples were further screened for the presence of IgM. If both IgM and IgG were positive, the endpoint titer of T1, T2, or T3 was determined. In cases with a fourfold increase in titer, real-time PCR of T1 samples was performed to detect *R*. *typhi* DNA. In total, 71/176 (40.3%) patients tested positive for IgG antibody, and 26 AFI cases were confirmed as MT (23 cases by PCR, 3 cases by fourfold titer increased IgG or IgM titer). The most common clinical symptoms in the confirmed cases were headache (80%), arthralgia (73%), malaise (69%), and myalgia (54%). In these cases, the presumptive clinical diagnoses were typhoid fever (43.2%), dengue (38.5%), and leptospirosis (19.2%). MT was not considered in any of the patients, and no patients received doxycycline. These findings confirmed that MT is an important cause of AFI in Indonesia. MT should be included in the differential diagnosis of AFI, and empirical treatment with doxycycline should be considered.

## Introduction

Murine typhus (MT) is a flea-borne disease caused by the obligate intracellular gram-negative bacteria *Rickettsia typhi* (*R*. *typhi*) [1]. Its life cycle requires vertebrate hosts, typically the rat, but humans can be incidentally infected. *R*. *typhi*-infected flea feces contaminate excoriated skin, leading to human infections, as the fleas bite also cause itching [2]. Clinical manifestations are generally non-specific, and *R*. *typhi* infection frequently manifests as an undifferentiated acute febrile illness (AFI) [3]. The diagnosis of MT can be made via serology or molecular diagnostics [4]. Several serological tests are available, including enzyme-linked immunosorbent assay (ELISA), microagglutination assay, and immunofluorescence assay (IFA). IFA remains the gold standard for serodiagnosis of MT; however, this test requires a fluorescence microscope and experienced technicians, which, like molecular diagnostics, is not available in many endemic resource-limited settings [5, 6]. Consequently, these tests are mostly restricted to specific research projects and are not available in routine laboratory services. Hence, there is a great need for point-of-care diagnostics to detect MT infection in limited healthcare settings.

MT occurs worldwide, but the prevalence differs widely. It has been reported as an important cause of febrile illness in different areas in Southeast Asia [7]. Still, the report of MT infections in humans in Indonesia is limited to two studies [2, 8], and in West Java, rodent infection was reported in one study [9]. A high proportion of MT cases were confirmed in a prospective cohort study of adult patients with AFI in Bandung, West Java [10], and the present report describes their clinical and laboratory features, as well as seroprevalence.

## Materials and methods

### Study design

The current study is part of a prospective cohort study of AFI patients conducted between July 2014 and February 2016 [10]. The parent study aimed to define the performance of IMS (Infection manager systems) tools equipped in hematology analyzer, to differentiate bacterial and viral diseases in Indonesia. In addition to that, the IMS was also tested in a large cohort of healthy Dutch adults [10]. Three hospitals in the great Bandung area and subdistricts, West Java province in Indonesia (Hasan Sadikin University Hospital and Salamun General Hospital, and Cibabat General Hospital), and two private practitioner clinics participated in this study. Hasan Sadikin General Hospital (HSH) is the main provincial tertiary referral hospital, while Salamun and Cibabat General Hospitals are secondary district hospitals. Bandung is the capital city of West Java Province, which lies at 400–791 m above sea level (asl). The southern part of the city consists mostly of sloping contours passed by 15 rivers and is prone to floods, while the northern part is far more mountainous. In 2014, Bandung was inhabitants of 2.575.478 in 16.729,65 hectares; the population density was 15.393 people/km^2^. Morbidity is mostly caused by infectious diseases due to unhealthy lifestyles, dense and slum settlements, and the high mobility of its people [11].

#### Study population

The inclusion criteria were subjects aged >14 years old presenting with an AFI and clinical suspicion or evidence of dengue, chikungunya, typhoid fever, leptospirosis, rickettsiosis, or any other common bacterial infection. Exclusion criteria were patients with suspected chronic infection, such as tuberculosis or HIV, severe concomitant conditions like dialysis, autoimmune diseases, malignancies, and pregnancy.

#### Study procedures

Patients meeting the study criteria were enrolled after receiving information regarding this study and providing written informed consent. Informed consent was obtained in parents’ or guardians’ presence in patients aged 14–18. Demographic data, medical history, physical examination, results of laboratory and radiology tests, and suspected diagnoses were recorded in a standardized electronic case report form. During hospitalization, patients were clinically evaluated, and additional diagnostic tests were performed as indicated. Also, patients visited the clinic 7–14 days after enrollment (T3). In hospitalized patients, blood was drawn at enrollment (acute sample, T1), three days thereafter (midterm sample, T2), and during follow-up on days 7–14 (convalescent sample, T3), while in outpatients, blood was drawn at enrollment (T1) and convalescent (T3). Four hundred sixty-three febrile patients were enrolled (S1 Fig), and a microbiological non-MT confirmation (dengue, leptospirosis, typhoid, chikungunya, malaria) was made in 164 cases (35.4%) (10). The remaining 299 AFI cases were presumed unknown, among which 176 had paired samples, T1 as well as T2 and or T3 samples. This group was further screened for MT (S1 Fig).

### Microbiological assays and case definition

IgG serology was performed on 176 convalescent (T3) or interim (T2) samples to determine seroprevalence. Sera were tested by ELISA typhus group rickettsiae (TGR) specific antibodies using *R*. *typhi* ELISA antigen preparations, respectively, following published procedures developed in-house by Richards et al. [12]. The positive IgG T2/T3 samples were then tested for *R*. *typhi* IgM to identify confirmed cases. Paired samples (T1 and T2/T3) of cases positive for both IgM and IgG antibodies underwent a further titration procedure to determine seroconversion or a fourfold increase in titer, and acute samples (T1) were subjected to molecular diagnostics (real-time PCR) [5]. Positive PCR samples underwent DNA sequencing targeting a 743-bp fragment of the outer membrane protein B (*ompB*) gene of *R*.*typhi*, and the sequence was deposited in GenBank and compared with other sequences using BLASTn. Sequences from the same species were downloaded for phylogenetic reconstruction analysis using the Maximum Likelihood method in MegaX [13]. The mapping of patient distribution based on the home address was performed using quantumGIS [14]. A confirmed MT case was defined when samples showed seroconversion or a ≥4-fold increase in titters between paired samples, as well as any patient sample with a positive PCR, irrespective of the serologic result screening [12]. For the screening procedure, we used a screening cutoff net OD value of 0.5 (1:100 dilution). For the titration procedure, a sample was considered positive if the sum net OD value of the four-fold dilutions was greater than 1.0 net absorbance. The end titer was subsequently determined to be the inverse of the highest dilution that gave a net absorbance of 0.2 or greater [9, 12].

### Statistical analysis

Descriptive statistics characterized clinical and laboratory profiles of confirmed cases. Categorical data were presented as numbers and percentages, while numerical data were presented as mean with standard deviation or median with interquartile range (IQR) or range. All statistical analyses were performed using STATA version 15.1 (Stata Corp, College Station, TX).

### Ethical statement

Ethical approval for this research was obtained from the Ethical committee Hasan Sadikin Hospital, Bandung, Indonesia, No.LB.04.01/A05/EC/070/IV/2014. Written informed consent was obtained from all study participants. For children participants, we obtained written informed consent from their parents or guardians.

## Results

### Serological testing and real-time PCR assay

**Fig 1:**
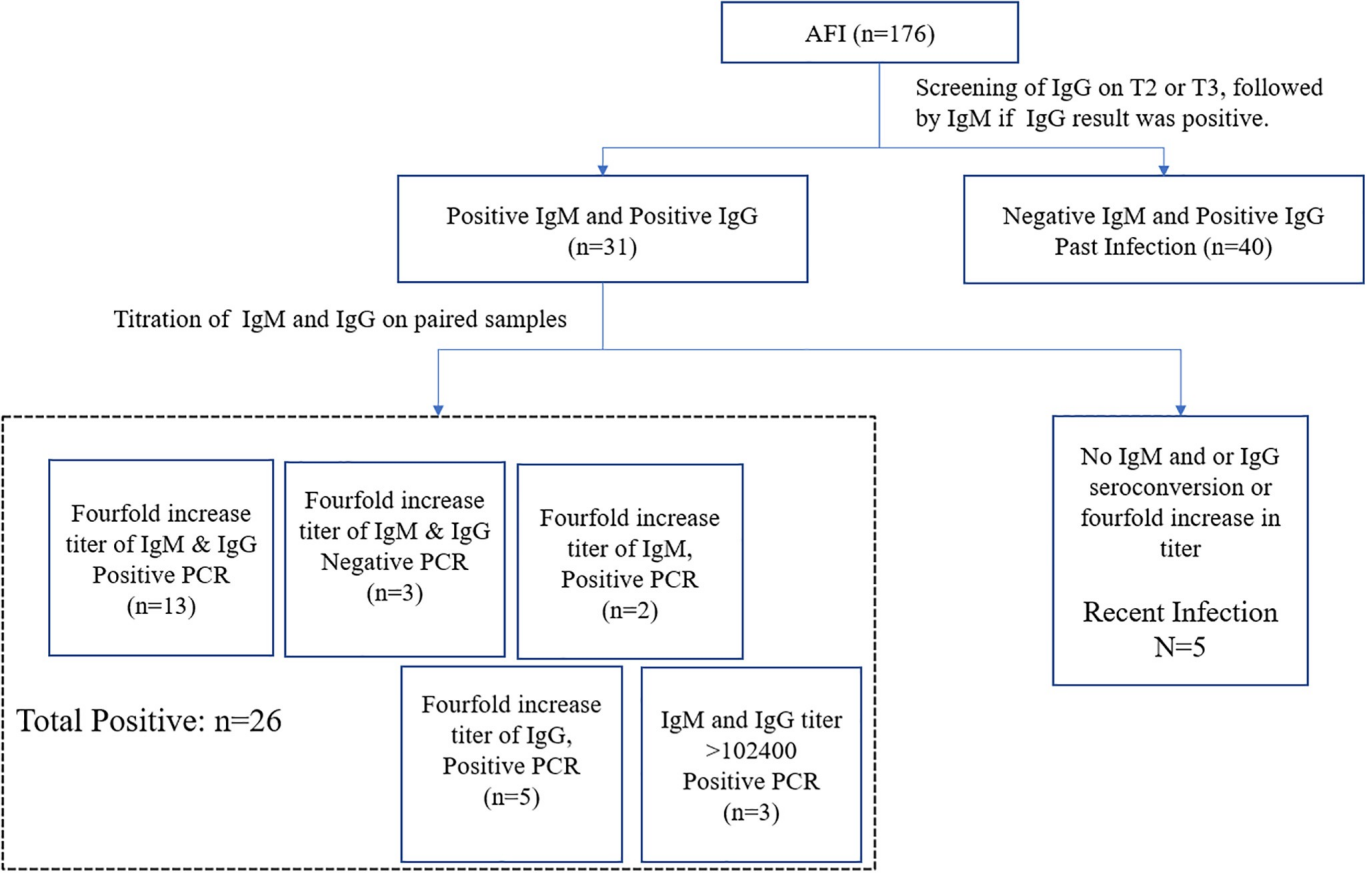
Overview of serological testing. Blood was collected from hospitalized patients at enrollment (acute sample, T1), day 3 (midterm sample, T2), and during follow-up on days 7–14 (convalescent sample, T3), while in outpatients, blood was drawn at enrollment (T1) and convalescence (T3).

Of the 176 samples tested, 71 (40.3%) were IgG-positive, of which 31/176 (17.6%) were positive for IgM, and 40/176 (22.7%) were negative for IgM, suggesting past infection. IgG and IgM titers were determined in 31 subjects with positive IgM and IgG serology. A fourfold or higher increase in anti-*R*. *typhi* IgG and or IgM titers were observed in 26 of these 31 cases (Fig 1).

**Fig 2:**
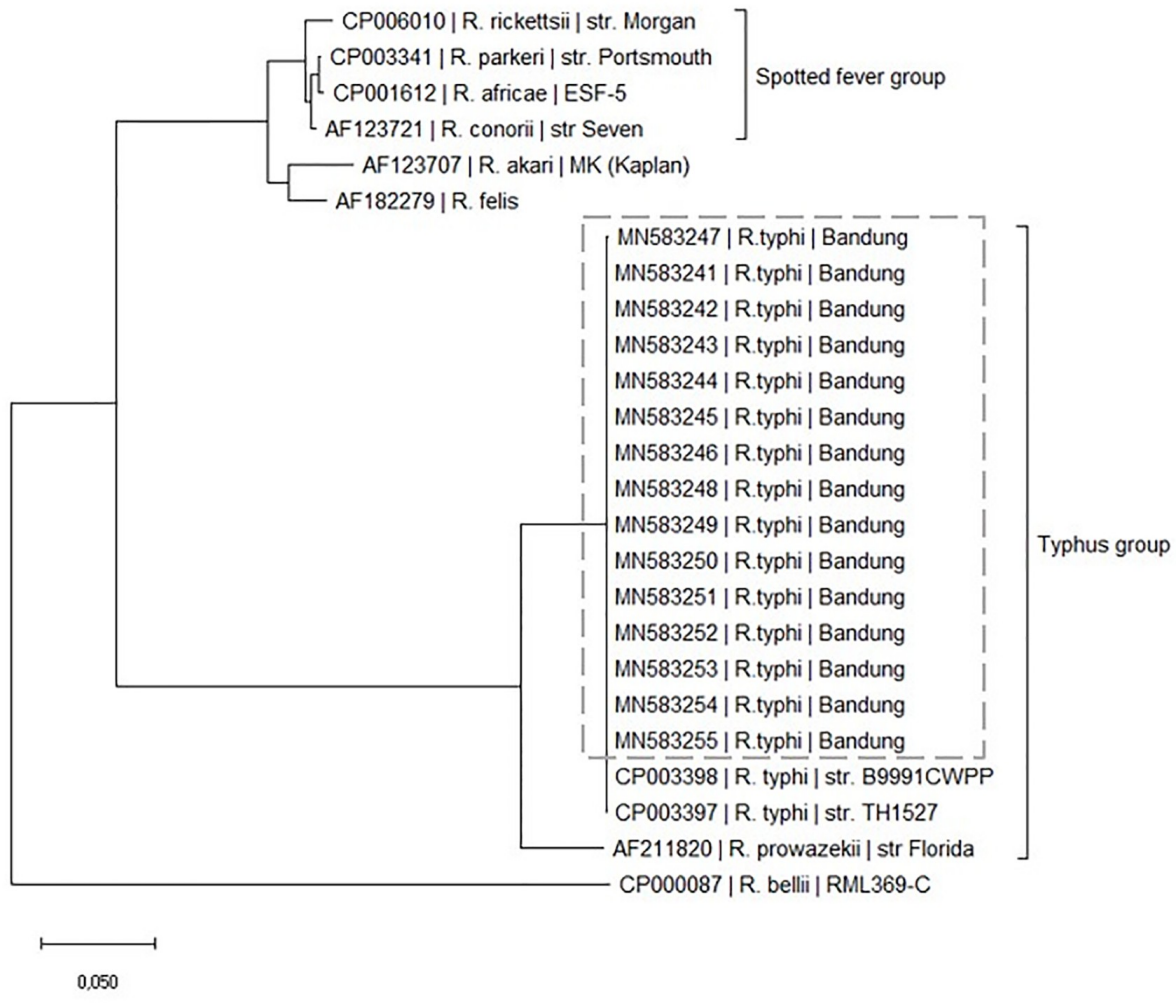
Phylogenetic tree of *R*. *typhi* outer membrane protein B (ompB) gene (704 bp; nucleotide 3556–4259) of the 15 MT cases (Genbank accession number MN583241 to MN583255). Evolutionary analyses were conducted using Mega X. All genomes were imported from GenBank NCBI (USA): http://www.ncbi.nlm.nih.gov/genome/.

Real-time PCR on the acute T1 sample was positive in 23/26 (88.4%) cases; 15/23 nucleotide sequences were available in GenBank (Accession number MN583241 to MN583255) (S2 Table), showing 100% homology with the reference strain *R*. *typhi* str. B9991CWPP (GenBank CP003398) from Myanmar and str.TH1527 (GenBank CP003397) from Thailand (Fig 2).

### Distribution and characteristics of MT cases

#### Patient distribution

All 26 confirmed MT cases were hospitalized in HSH (11/80 patients, or 13.8%), Salamun Hospital (5/34 patients, or 14.7%), and Cibabat Hospital (10/62 patients, or 16.1%). Patients originated from Bandung city and more rural settings.

#### Characteristics of MT cases

Confirmed MT cases’ median age was 33.5 years (IQR 23.3–50.8, range 17–61), and 69% were male. The median of fever onset before admission was seven days (range 1–13), and the type of fever was continuous in 7 patients (27%), intermittent in 4 (15.4%), and remittent in 15 (57.7%) cases. There was a wide variety of clinical symptoms and signs, but most were non-specific (Table 1). Common symptoms were gradual onset fever, headache (81%), and arthralgia (73%). The typical triad of rickettsioses (fever, headache, and rash) was observed in only two (8%) cases. Physical examination revealed a few abnormalities, such as hepatomegaly (n = 2) or jaundice, conjunctival suffusion (n = 3), or skin rash (n = 3). Routine laboratory tests were also mostly non-specific. Blood counts showed normal hemoglobin levels (20/26, 77%) and thrombocytopenia (21/26, 80%). Leucopenia and leukocytosis were observed in 3/26 (11.5%) and 5/26 (19.2%) patients, respectively. A liver function test was performed on six subjects, revealing mildly increased SGOT/AST and bilirubin (direct and indirect) levels. The median (IQR) concentrations of C-reactive protein (CRP) and procalcitonin were 76.2 mg/L (50.5–127.3) and 1.7 units (1.0–4.8), respectively (Table 1). In all cases, MT was not suspected clinically by the treating physician. Presumed diagnoses at admission were typhoid fever (42.3%), dengue (38.5%), and leptospirosis (19.2%). In all 26 confirmed cases, T2 or T3 were negative for IgM anti-dengue, and T1 samples were negative for RT-PCR flavivirus and dengue nonstructural protein 1 (NS1). Ten T2 or T3 samples were positive for IgM anti-leptospira, but no leptospira DNA was detected in T1 samples by PCR. Seven T2 or T3 samples were positive for IgM anti-Salmonella typhi, but their T1 samples were negative for blood culture. Fifteen confirmed MT patients received antibiotics, but none received doxycycline (S1 File).

**Table 1:** Patient characteristics, clinical and laboratory data of confirmed MT cases.

Characteristic	Confirmed MT (N = 26)
**Symptoms**	
Fever	
• Fever duration in days (median, range)	7 (1–13)
• Gradual onset of fever (n, %)	18 (69.2%)
• Continuous fever (n, %)	7 (26.9%)
Other symptoms	
• Triad Rickettsiosis: fever, headache, and rash (n, %)	2 (8%)
• Headache (n, %)	21 (81%)
• Abdominal pain (n, %)	17 (65.5%)
• Malaise (n, %)	18 (70%)
• Arthralgia (n, %)	19 (73.1%)
• Myalgia (n, %)	14 (53.8%)
• Skin rash (n, %)	3 (11.5%)
**Outcome (upon discharge)**	
• Recovered (n, %)	19 (73.1%)
• Recovered with sequelae (n, %)*	1 (3.8%)
• No improvement (n, %)	1 (3.8%)
• No Data (n, %)	5 (19.2%)
**Laboratory examinations**	
• Hemoglobin (g/dl) (median, IQR)	13.4 (12.7–14.5)
• Hematocrit (%) (median, IQR)	38.0 (36.0–40.3)
• Platelet count (x10^3^/μL) (median, IQR)	85.5 (68.0–125.0)
• Thrombocytopenia (<150 x10^3^/μL) (n, %)	21 (80.1%)
• Leukocyte count (x10^3^/μL) (median, IQR)	7.6 (5.8–10.3)
• Leukopenia (<5 x10^3^/μL) (n, %)	3 (11.5%)
• Leukocytosis (>11 x10^3^/μL) (n, %)	5 (19.2%)
• Thrombocytopenia and normal leucocyte (n, %)	14 (53.8%)
• Neutrophils (%) (median, IQR)	74.4 (63.0–81.7)
• Lymphocytes (%) (median, IQR)	16.5 (13.0–30.2)
• Neutrophil to lymphocyte count ratio (median, IQR)	4.6 (2.1–6.0)
• CRP, unit (median, IQR)	76.2 (50.7–127.4)
• Procalcitonin, unit (median, IQR)	1.7 (1.1–4.8)
• SGOT/AST (U/L) (median, IQR) (n = 6)	196 (62.0–270.0)
• SGPT/ALT (U/L) (median, IQR) (n = 6)	136 (64.0–225.0)
• Ureum (mg/dl) (median, IQR) (n = 10)	39.0 (24.0–45.0)
• High creatinine (> 1.3 mg/dl) (n, %) (n = 10)	1 (10%)
• High total bilirubin (> 1.0 mg/dl) (n; %) (n = 5)	5 (100%)

*Lethargy

## Discussion

This study shows that acute *R*. *typhi* is a common cause of acute febrile illness among hospitalized adult patients in West Java. The MT patients in this study came from urban and rural areas, suggesting that it is highly endemic in the region. Furthermore, MT was the third most common confirmed infection (after dengue and commensal bacterial infections) in this cohort [10]. Remarkably, the diagnosis was not considered by any of the treating physicians, and the preferred recommended therapy (doxycycline) was not administered.

The seroprevalence among hospitalized fever patients in the greater Bandung area (40.3%) was similar to another report of the seroprevalence in Bandung city (37.7%) (3), confirming that *R*. *typhi* is an important infectious agent to be considered when managing patients with AFI in Indonesia [8].

Our screening method for IgM and IgG antibodies, followed by examining for seroconversion or increased titer in positive samples, yielded a high number of cases (~74%) with *R*. *typhi* DNA. The sensitivity of *R*. *typhi* DNA PCR has been reported to be lower when samples are only screened using IgM (33%) [15] or only screened using IgG (3.3%) [16]. Residual antibodies from previous infections also cause difficulty in the confirmation using only serological methods [15, 17]. Thus, in endemic areas, both IgM and IgG serology and PCR should be performed upon admission of suspected MT cases.

The genetic makeup of our cases was similar to those reported in Thailand (*R*. *typhi* strain TH1527), the first human case in Thailand in April 1965 (GenBank accession number CP003397), and Myanmar (*R*.*typhi* strain B9991CWPP), which was isolated from *Bandicota* rats (*Bandicota sp*.) in the 1970s (GenBank accession number CP003398) [18]. Rodents are important reservoirs for MT in Indonesia, and high MT seropositive rodents have been reported on Java Island [9, 19]. *Xenopsylla cheopis*, *the* main rat-flea vector of MT, also harbors *R*. *felis* [20*]*, although no human infected with *R*. *felis* has been documented in Indonesia.

Despite the fact that several studies from Indonesia have reported a substantial number of MT cases [2, 4, 21–23], healthcare workers were unaware of the high proportion of MT among patients with AFI. The lack of a laboratory test to diagnose MT might be attributed to this. The importance of MT among patients with fever has been reported previously when the Weil Felix test was in place [12]. Patients with MT lack specific clinical symptoms, while the rash, which is only present in 11% of cases in this study, can easily be missed. The clinical presentation is similar to other common AFI, especially dengue, typhoid fever, and sometimes leptospirosis, which were more often considered by residing physicians [8]. While the hematological profiles in *R*. *typhi* cases were similar to typhoid, they were slightly different from dengue and leptospirosis. Dengue often showed higher hemoglobin, lower leukocyte, and platelet counts, while leptospirosis showed higher leukocyte and neutrophil counts but lower absolute lymphocyte counts [21]. Although increased total bilirubin and direct bilirubin have been reported in MT cases, these phenomena were more common in dengue and typhoid. In contrast, the increased total and indirect bilirubin were more frequent in leptospirosis [21]. Diagnosis depends on high awareness among healthcare professionals and biomarker (high CRP result) analysis with a specific microbiological test. The current gold standard of MT diagnosis relies on the fourfold increase or seroconversion of serology using an immunofluorescent assay or ELISA, which is not widely available as specialized laboratory equipment is necessary [5]; therefore, there is an urgent need for the development of point of care tests.

In this study, the clinical course of patients was mostly mild without any fatalities, which is different from another report from Indonesia reporting mortality among MT cases of 7/102 (6.8%) [8]. Severe illness with pulmonary or CNS involvement, kidney failure, hepatitis, or multiorgan failure has also been documented [8, 24]. In this study, no patients received doxycycline, although the recovery was complete in 19/26 patients. Doxycycline (100 mg bd) is the drug of choice for MT [25]. It has been reported to shorten the fever duration, especially when given early [26]. The alternative treatment options include chloramphenicol and quinolones (ciprofloxacin, levofloxacin) [27, 28]. The mean time to defervescence was 2.9 days for doxycycline, 4.0 days for chloramphenicol, and 4.2 days for ciprofloxacin [26]. Azithromycin is not effective compared to doxycycline for murine typhus therapy [29].

This study has some limitations; importantly, not all samples could be evaluated by molecular techniques; thus, some early acute cases with no convalescence samples might have been missed. Furthermore, it was not designed to be a specific study for rickettsia; thus, it lacked complete follow-ups and rickettsia-specific clinical features, such as proper examination for possible skin lesions and no screening for another rickettsial disease, such as the scrub typhus or epidemic typhus, is being performed. Currently, epidemic typhus has never been reported in Indonesia. Several serosurveys have reported the detection of antibodies against scrub typhus from community households and febrile cases [12, 21], but acute human cases have never been reported in Indonesia. However, the complete enrollment of AFI patients in this study did show the etiological spectrum and proportion of MT compared to other etiologies. The incomplete set of samples in this study highlights the need for meticulous planning in developing a future fever cohort study, as the patients are often reluctant to have their blood taken in the convalescent phase when they are feeling well. Finally, co-infections with other pathogens are possible; as in the parent study [10], no further testing for MT was performed when cases were already assigned to an etiology. Furthermore, the presence of co-infections may be underdiagnosed, as gold standard diagnostic tests were not used, which would include, for instance, leptospirosis microscopic agglutination test (MAT) or urine culture, or PCR. Still, to the best of our knowledge, co-infection between MT and other diseases has never been reported in Indonesia, suggesting a low possibility of co-infections.

## Conclusions

In conclusion, murine typhus is endemic in Indonesia, and the diagnosis should be considered in patients with an acute febrile illness resembling typhoid, leptospirosis, or dengue. Doxycycline is the drug of choice, which may also be used in patients with leptospirosis, an essential alternative diagnosis. Point-of-care tests for MT are urgently needed for broader access to diagnosis. Better strategies are required for the prevention, including the reduction of the reservoir and limiting transmission.

## Supporting information

S1 Fig Diagnostic algorithm to identify Rickettsia infections. The microbiological tests in the parent study [10] included blood cultures, dengue NS1 rapid test, paired dengue IgM and IgG serology and dengue RT-PCR, RDTs or serology for chikungunya IgM, Salmonella IgM, and Leptospira IgM followed by a specific serum or whole blood PCRs for these pathogens. The remaining cases without a proven diagnosis were tested for *Rickettsia typhi*.
(TIF)

S1 Table Definitions for categorizing clinical manifestations. (DOCX)

S2 Table Murine typhus confirmatory results in confirmed cases. (DOCX)

S1 File Murine typhus in Bandung_Sept2022. (XLSX)
