# Supplementary figures and images for: Murine typhus is a common cause of acute febrile illness in Bandung, Indonesia
Source: PLoS One. 2023 Jul 7;18(7):e0283135. doi: 10.1371/journal.pone.0283135 (PMC10328256; doi:10.1371/journal.pone.0283135)

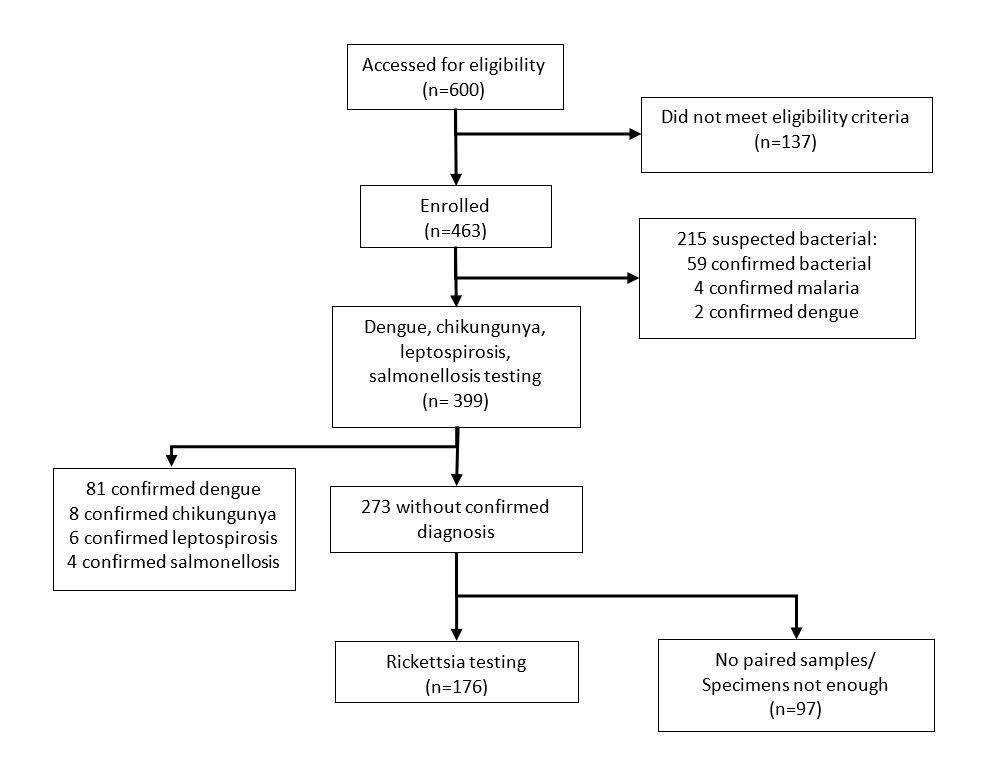

Supplement: S1 Fig — The microbiological tests in the parent study [10] included blood cultures, dengue NS1 rapid test, paired dengue IgM and IgG serology and dengue RT-PCR, RDTs or serology for chikungunya IgM, Salmonella IgM, and Leptospira IgM followed by a specific serum or whole blood PCRs for these pathogens. The remaining cases without a proven diagnosis were tested for Rickettsia typhi. (TIF) [file pone.0283135.s001.tif]
