# Supplementary material for: Murine typhus is a common cause of acute febrile illness in Bandung, Indonesia
Source: PLoS One. 2023 Jul 7;18(7):e0283135. doi: 10.1371/journal.pone.0283135 (PMC10328256; doi:10.1371/journal.pone.0283135)
Supplement: S1 Table — (DOCX) [file pone.0283135.s002.docx]

**S1 Table. Definitions for categorizing clinical manifestations**

| **Signs/Symptoms** | **Description** |
| --- | --- |
| Fever | Temporary rise in body temperature as measured by axillary temperature (≥37.5ºC or 99.5 ºF) |
| Continuous Fever | Continuous or sustained fever is defined as fever that does not fluctuate more than about 1 °C (1.5 °F) during 24 h, but at no time touches normal. |
| Intermittent | Intermittent fever is defined as fever present only for several hours during the day. |
| Remittent | Remittent fever is defined as fever with daily fluctuations exceeding 2 °C but at no time touches normal. |
