# Supplementary material for: Murine typhus is a common cause of acute febrile illness in Bandung, Indonesia
Source: PLoS One. 2023 Jul 7;18(7):e0283135. doi: 10.1371/journal.pone.0283135 (PMC10328256; doi:10.1371/journal.pone.0283135)
Supplement: S2 Table — (DOCX) [file pone.0283135.s003.docx]

**S2 Table. Murine typhus confirmatory results in confirmed cases**

| **Case ID** | **Realtime PCR (T1)** | **GenBank Acc No.** | **IgM results**  **(T1–T2/T3)** | **IgG result**  **(T1-T2/T3)** |
| --- | --- | --- | --- | --- |
| MT01 | Positive |  | >102400* | >102400* |
| MT02 | Positive | MN583244 | ≥4x | ≥4x |
| MT03 | Positive | MN583245 | ≥4x | 25600* |
| MT04 | Positive |  | ≥4x | ≥4x |
| MT05 | Negative |  | ≥4x | ≥4x |
| MT06 | Positive | MN583255 | >102400* | ≥4x |
| MT07 | Positive |  | ≥4x | ≥4x |
| MT08 | Positive | MN583246 | >102400* | ≥4x |
| MT09 | Positive | MN583243 | ≥4x | ≥4x |
| MT10 | Negative |  | ≥4x | ≥4x |
| MT11 | Positive |  | ≥4x | ≥4x |
| MT12 | Positive | MN583248 | ≥4x | ≥4x |
| MT13 | Positive |  | ≥4x | ≥4x |
| MT14 | Positive |  | ≥4x | ≥4x |
| MT15 | Positive | MN583247 | >102400* | >102400* |
| MT16 | Positive | MN583251 | ≥4x | ≥4x |
| MT17 | Positive | MN583249 | 6400* | ≥4x |
| MT18 | Positive | MN583250 | >102400* | >102400* |
| MT19 | Positive |  | ≥4x | ≥4x |
| MT20 | Positive | MN583252 | >102400* | ≥4x |
| MT21 | Positive | MN583253 | ≥4x | ≥4x |
| MT22 | Positive | MN583254 | ≥4x | ≥4x |
| MT23 | Positive |  | ≥4x | ≥4x |
| MT24 | Positive | MN583241 | >102400* | ≥4x |
| MT25 | Positive | MN583242 | ≥4x | >102400* |
| MT26 | Negative |  | ≥4x | ≥4x |

*Titer in T1 and T2/T3
